# Ranking Technologies of Additive Manufacturing of Removable Complete Dentures by the Results of Their Mechanical Testing

**DOI:** 10.3390/dj11110265

**Published:** 2023-11-13

**Authors:** Dmitry I. Grachev, Igor V. Zolotnitsky, Dmitry Yu. Stepanov, Alexander A. Kozulin, Magomet Sh. Mustafaev, Aslan V. Deshev, Dmitriy S. Arutyunov, Islam V. Tlupov, Sergey V. Panin, Sergey D. Arutyunov

**Affiliations:** 1 Digital Dentistry Department, A.I. Yevdokimov Moscow State University of Medicine and Dentistry, 127473 Moscow, Russia; grachev@msmsu.ru (D.I.G.);; 2 Orthopedic Dentistry Department, A.I. Yevdokimov Moscow State University of Medicine and Dentistry, 127473 Moscow, Russia; zolotnitskiy@msmsu.ru; 3 Laboratory of Mechanics of Polymer Composite Materials, Institute of Strength Physics and Materials Science of Siberian Branch of Russian Academy of Sciences, 634055 Tomsk, Russia; sdu@ispms.ru; 4 Department of Solid Mechanics, Physical-Technical Faculty of National Research Tomsk State University, 634050 Tomsk, Russia; kozulyn@ftf.tsu.ru; 5 Institution of Dentistry and Maxillo-Facial Surgery, Kabardino-Balkarian State University Named after H.M. Berbekov, 360004 Nalchik, Russia; musmag@kbsu.ru (M.S.M.); a.v.deshev@kbsu.ru (A.V.D.); i.v.tlupov@kbsu.ru (I.V.T.); 6 Propaedeutics and Prosthodontics Technology Department, A.I. Yevdokimov Moscow State University of Medicine and Dentistry, 127473 Moscow, Russia; arutyonov-ds@msmsu.ru

**Keywords:** complete removable denture, polymer testing, mechanical properties, finite element method, decision making, additive manufacturing, polymethylmethacrylate, ranking, analytic hierarchy process

## Abstract

In this study, a methodology was developed for ranking manufacturing technologies of removable complete dentures (RCDs) according to the results of their full-scale mechanical tests. The actuality of the study is motivated by establishing the advantages and drawbacks of 3D-printed RCDs in contrast with ones manufactured via an analog protocol. The RCDs were fabricated via four technological routes that included various combinations of subtractive technologies (hot polymerization/HP and CAD/CAM milling) and additive manufacturing (digital light processing/DLP) ones and the installation of commercially available cosmetic denture teeth (DT). In the mechanical tests, different blocks of teeth (incisors, canines, premolars and molars) were loaded. To solve the ranking problem, it was proposed to interpret the results of the mechanical tests in terms of the reliability, durability and compliance/stiffness criteria. For this purpose, the combined AHP-VIKOR method was applied. In addition, a computer simulation of the mechanical loading conditions and the response of the RCDs was performed based on the finite element method (FEM). As the key conclusion, it was stated that additive manufacturing (AM) methods are competitive and cost-effective techniques for the fabrication of RCDs.

## 1. Introduction

Despite the widespread implementation of numerous methods of dental orthopedic treatment of edentulous patients, removable complete dentures (RCDs) are still in high demand. The problem is associated with both age-related changes of the mouth tissues, as well as complications of caries and periodontal diseases [1]. For such patients, prosthetics is a complex medical procedure, often characterized by ineffective results [2]. The main reasons to apply for repeated prosthetics are mobility, poor retention and stabilization of prostheses and the presence of pores, deep scratches and cracks in denture bases, which cause their failure [3].

The development of various additive manufacturing (AM/3D printing) methods has opened up broad prospects for rapid and cost-effective planning and subsequent treatment of edentulous patients. In such cases, polymethyl methacrylate (PMMA) is the most common feedstock, which is also used for 3D printing of both denture bases and dentitions [4]. In a previous paper by the authors [5], several brands of commercially available PMMA, applied in digital light processing (DLP) technology, were studied and ranked. However, in addition to the technological aspects of manufacturing RCDs, it is important to evaluate their effectiveness as a dental structure in terms of deformation behavior under applying point loads. In this formulation, an important role is assigned to methods of computer simulation of deformation processes, including those under cyclic loading [6]. Nevertheless, conducting full-scale experiments is relevant to verify models as well as to design dental structures. In this case, two options are possible: (i) AM of a denture base with the installation of cosmetic or CAD/CAM milled denture teeth or (ii) 3D printing of an entire dental structure [7].

AM of RCDs is based on the development of a digital model in which a dentition is designed by an operator or dental technician, i.e., where configurations (including size, articulation of adjacent teeth, their adjacency to a denture base, etc.) are proposed from general ideas or a database) about (i) dimensions and shapes of different types of teeth; (ii) anatomical features of the oral cavity (alveolar ridge) of a particular patient; and (iii) programming the occlusion of antagonist teeth. In this regard, the importance of computer simulation of the loading processes of an RCD is rising, in particular by applying algorithms based on the finite element method (FEM). At the same time, experimental verification of the computer simulation results should be performed using the methods of experimental mechanics, where a controlled point load is applied to a specific tooth or block of teeth [8].

In such cases, it is essential to compare the advantages and drawbacks of 3D-printed RCDs, as well as to modify the dentition design or its articulation with a denture base (if necessary). An important aspect is to ensure the stability of both individual teeth in the structure and the entire prosthesis on an alveolar ridge. Under compressive loads, individual teeth can lose stability (from the strength of materials point of view). On the other hand, RCDs possess inconstant fixation conditions when placed on the alveolar ridge, changing the pattern of stress–strain states in its various parts. As a result, both the denture base and individual RCD parts can be fractured even under the application of static loads.

In addition to the experimental verification of the FEM-based computer simulation results, a significant amount of data is registered during full-scale tests that require proper processing, analysis and interpretation. In this regard, one should highlight the particular mechanical properties of the RCD, in order to rank the data over the importance order. Then, multicriteria optimization algorithms should be applied considering the obtained results [9,10].

In the aspect of developing digital twins, it is important for a dentist to make a fact-based selection of a prosthesis design, taking into account (i) individual morphological features of edentulous jaws, (ii) an appropriate feedstock for manufacturing an RCD and, most importantly, (iii) a production route adequate for these conditions. The correct combination of these variables is the key to effective RCD treatment of such patients. 

Based on the above, the aim of the study was to develop a methodology for ranking digital methods for manufacturing RCDs according to the results of their full-scale mechanical tests. To achieve it, deformation responses were examined for RCDs fabricated using advanced both subtractive and AM methods. The ‘analog protocol’ included a combination of hot polymerization (HP) of the denture base with installed cosmetic denture teeth (DT), while the full or partial ‘digital protocol’ comprised the DLP for (i) both the denture base and dentition; (ii) the denture base with installed cosmetic DT; and (iii) a denture base with installed CAD/CAM milled teeth. In these cases, different blocks of teeth (incisors, canines, premolars and molars) were loaded.

The paper is structured as follows. In Section 2, the research objects are characterized, namely, full-scale RCD samples made from PMMA using the subtractive and AM technologies, and, as well, their testing methods are described. Section 3 presents the results of the deformation response evaluation (in the elastic region) for the RCDs via point loading on each block of teeth. In Section 4, the criteria for assessing the quality of prostheses based on the above characteristics are reported, and the production routes for the RCDs are ranked. Section 5 is devoted to a computer simulation of the RCD loading process, considering, to a certain extent, the obtained experimental results.

## 2. Materials and Methods

RCDs were fabricated using the production routes presented in Table 1. The choice of their factory-fabricated parts (cosmetic denture teeth/DT) was determined by their presence in the database of the EXOCAD 3.0 software package (Exocad GmbH, Darmstadt, Germany), which was applied for digital modeling of the RCDs.

No. 1 is ‘HP+DT’. The ‘Belacryl-M GO’ hot-cured PMMA (‘VladMiVa’ LLC, Belgorod, Russia) was used for the fabrication of the RCD base. Polymerization was carried out in a gypsum cuvette according to the following schedule: heating up to 90–100 °C for one hour followed by exposure at this temperature for one hour and subsequent slow cooling. Cosmetic (PMMA-based) DT is represented by the following composition: 40–60 wt.% PMMA; 20–30 wt.% quartz glass; and 10–20 wt.% a mixture of isomers of ure-tandimethacrylate (UDMA; ‘Gebdi Dental Products’, Yeti Dentalprodukte GmbH, Engen, Germany).

No. 2 is ‘3D+3D’. According to a single digital model, for which a dentition similar in design to cosmetic DT was taken from the digital library, the ‘3D+3D’ RCD was made using the DLP method (as a variation of the SLA technique). The polymerization degree was determined by the effect of UV radiation on PMMA, and the dentition material differed from the denture base one in having a higher elastic modulus. According to common practices, post-build processing (subsequent curing) was applied to promote cross-linking [12]. As noted above, the ‘3D+3D’ RCD was characterized by the presence of continuous transitions between adjacent teeth (as well as their monolithic pairing with the denture base).

No. 3 is ‘3D+DT’. The PMMA base was made using the same digital model, on which indentations were formed upon 3D printing for the subsequent installation of cosmetic denture teeth. The cosmetic DT attachment technology was identical to the ‘HP+DT’ RCD. An advantage of this approach was the use of cosmetic denture teeth of higher strength and wear resistance. A drawback was a prolonged duration of ‘manual’ setting and fastening of the dentition from a cosmetic DT set.

No. 4 is ‘3D+CAM’. The properties of PMMA blanks supplied for digital (CAD/CAM) milling were comparable to those for the cosmetic DT procedure, but a wider range of shapes and sizes of the dentition elements could be fabricated. In this case, the manufacturing technology of the denture base did not differ from those applied in the ‘3D+DT’ manufacturing route. The dentition elements were attached to a denture base in the same way as in the cosmetic DT procedures (production routes No. 1 and 3). It should be noted that the mechanical properties and their consistency for the CAD/CAM dentition were assumed to be high.

For AM of the RCDs, an ‘Anycubic Photon Mono X’ (Hongkong Anycubic Technology Co., Limited, Hong Kong, China) 3D printer was deployed. The ‘NOLATEK 3D LCD/DLP (pink)’ and ‘NOLATEK 3D LCD/DLP’ PMMA feedstocks (“VladMiVa” LLC, Belgorod, Russia) with flexural moduli of 1.4 and 1.6 GPa, respectively, were used to make the denture base and dentition.

The ‘Vladmiva NOLATEK2–BLOCK (for permanent crowns)’ material (‘VladMiVa’ LLC, Belgorod, Russia) was used for CAD/CAD milling of denture teeth with an ‘Arum 5X-400′ machine (ARUM dentistry, Daejeon, Korea).

**Figure 1:**
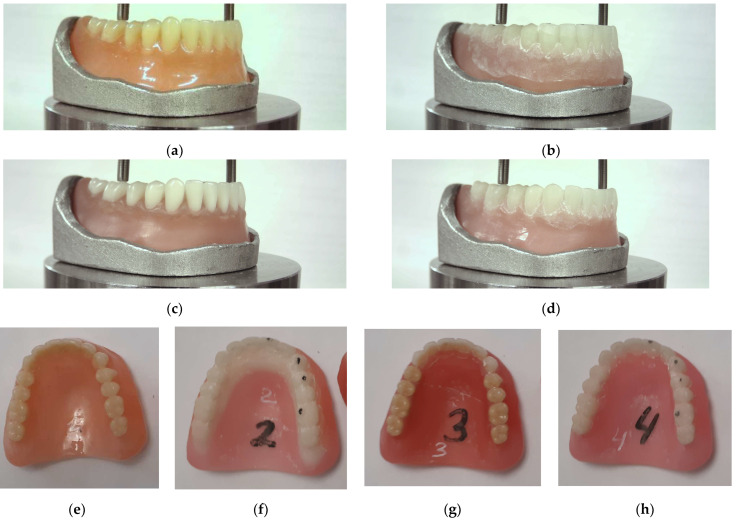
The photographs of all four types of the studied RCDs (the test mandrel (**a**–**d**) and bottom view (**e**–**h**)): (**a**,**e**) ‘HP+DT’; (**b**,**f**) ‘3D+3D’; (**c**,**g**) ‘3D+DT’; and (**d**,**h**) ‘3D+CAM’.

A more detailed description of the production routes for manufacturing the RCDs according to the analog protocol is given in [13]. All types of the studied RCDs were made using an identical digital template (STL model) in order to minimize the influence of possible deviations in dimensions and design on the test results. In the ‘3D+3D’ case, the articulation of adjacent teeth was smoother (compared to individually installed cosmetic DT) due to the specifics of their fabrication via the DLP method. Figure 1 shows photographs of all four types of the studied RCDs.

**Figure 2:**
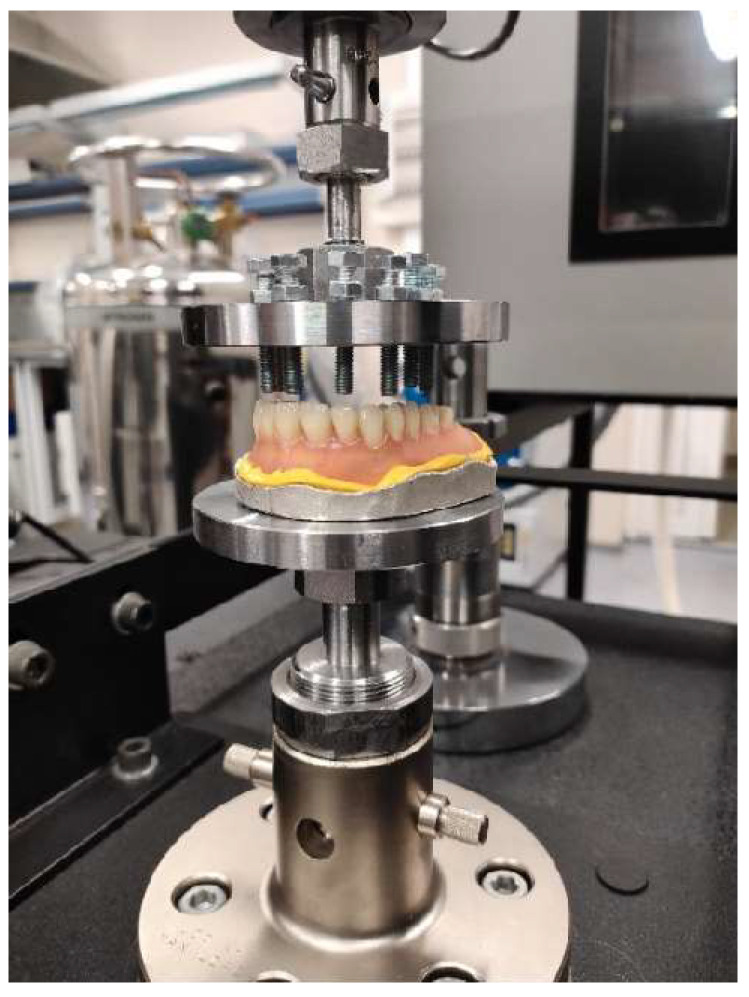
The Mechanical test fixture with an RCD sample.

In contrast to the rather non-rigid fastening of RCDs on the patient’s alveolar ridge, they were rigidly fixed on a metal foundation during the laboratory mechanical tests, which excluded their movement or distortion (Figure 2). An ‘Instron^®^ 5965′ electromechanical machine (Illinois ToolWorks Inc., Glenview, IL, USA) was used, equipped with a force gauge with a maximum force capacity of 5 kN (the force measurement accuracy is ±0.4% of a reading, down to 1/100 of load cell capacity). The machine also included two round platforms: the upper ‘loading’ one on its movable grip and the lower ‘support’ platform on the fixed grip. The ‘loading’ part was designed and manufactured in such a way that it enabled the selective loading of the RCDs at one or several points. The ‘reference’ part of the equipment was a platform for the installation of both upper and lower RCDs. In this study, the authors described the results of testing the maxillary RCDs only. Since both the RCDs and the platforms for their attachment were made on the basis of the same digital model, no additional fastening of the tested samples was required.

**Figure 3:**
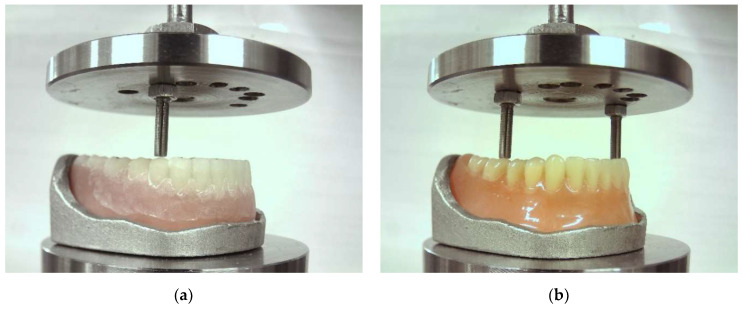
The fixtures prepared for testing the RCDs: (**a**) asymmetrical to the ‘3D+3D’ right premolar; (**b**) symmetrical on both ‘HP+DT’ molars.

Since the mechanical behavior of the RCD samples was studied, including for comparison with the computer simulation results, a load was applied on each of the blocks of teeth. Thus, all RCDs were tested in eight different variants: single incisor (V1), canine (V2), premolar (V3) and molar (V4) on one side (Figure 3a) and symmetrically (VV1, VV2, VV3 and VV4, respectively) on the other side (Figure 3b). The maximum load in each test reached 100 N (corresponding to a typical mastication level).

The preparation for testing the RCDs included several operations:

- Installation of a model prosthesis on the (lower) support platform;
- Installation of a loading element (a screw with a diameter of 3 mm and a length of 40 mm), or two similar ones under two-point loads, in the corresponding holes made in advance in the upper part of the equipment;
- Accurate positioning of the loading elements at the corresponding points.

The fixtures prepared for testing the RCDs are shown in Figure 3.

In the case of the two-point (symmetrical) loading on both sides of the RCDs, additional special attention was paid to ensuring symmetry at the preparation stage (in order to avoid misalignment). This was achieved in the following way: the upper grip was lowered until one of the two elements contacted the sample after precise positioning of the loading elements over the contact points (the developed force was equal to the preliminary load of 100 N). Then, the loading element was raised by 0.1 mm, and a gap with a tooth at the loading point was adjusted using a calibration plate 0.1 mm thick as a reference. 

After the preparation procedures, the tests began. Regardless of the current configuration of the loading elements, the RCDs were loaded up to the maximum level of 100 N, after which the upper part of the tool automatically returned to its initial position. The ∆*l* displacement values (in millimeters) were measured with a uniform time step. The procedure was repeated at least three times.

The criterion for the successful completion of the tests was a change in the maximum slope of the elastic region of a load–displacement diagram in the last test by less than ±10 N/m from the average value in the last three passes. This fact indicated that the loading conditions were completely identical (reproduced), but this test was not accompanied by the development of irreversible (plastic) strains. Those load–displacement diagrams were considered as the final ones, since the RCDs were in the most equilibrium state with respect to the loading element and the base platform upon testing.

Before reporting the obtained data, the authors considered it appropriate to formulate the problem statement from the standpoint of comparing the results of the mechanical tests.

The analog protocol for manufacturing the RCDs is associated with significant time costs (including waiting for a patient) and manual labor of a dental technician [14]. However, it also has a number of distinct advantages. Firstly, cosmetic denture teeth possess guaranteed high mechanical (strength and wear resistance) and aesthetic (color and polishability) properties that do not change from batch to batch. At the same time, the non-stationarity and high efficiency of manufacturing a dentition can cause some inconsistency in 3D printing, primarily in the mechanical characteristics (for example, due to incomplete polymerization, the formation of discontinuities, etc.). Secondly, a denture base is fabricated via hot polymerization under stationary conditions as well. This fact minimizes the possibility of the formation of discontinuities, warping, residual stresses, etc., if all technology regulations are met. Thirdly, before installing cosmetic denture teeth on a base, their additional preparation (surface activation) is carried out in order to increase the adhesion. Additionally, when the DT are installed, they are fixed by a hot-curing polymer in a plaster cuvette, which eliminates their displacement and contributes to good fixation. Conversely, denture teeth are installed on a 3D-printed base (fabricated by the DLP) in the ‘3D+DT’ and ‘3D+CAM’ cases, so a secure fixation in a virtually single dentition may not be achieved.

Thus, the ‘HP+DT’ RCD, manufactured according to the analog protocol, should possess improved strain–strength properties and can be taken as a ‘reference’ for comparison with other ones made via AM (partial or complete).

## 3. Experimental Results

### 3.1. The Mechanical Tests

**Figure 4:**
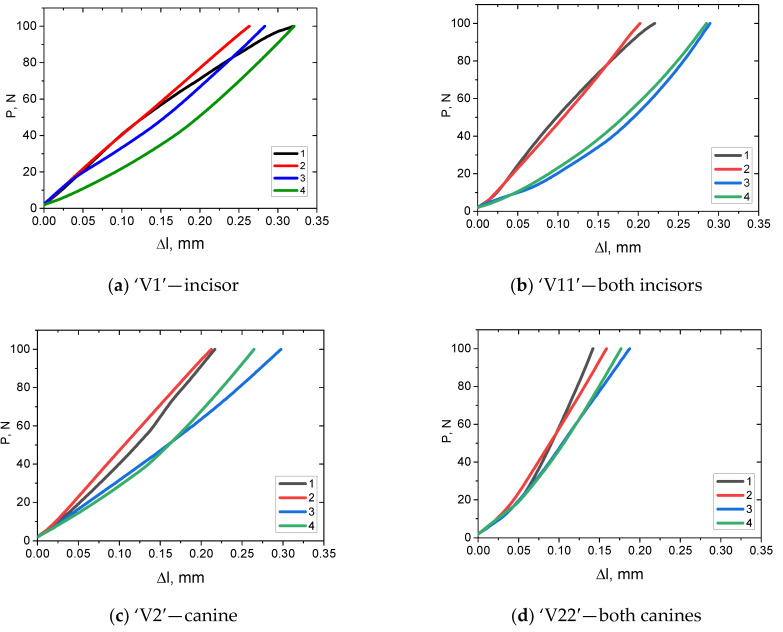

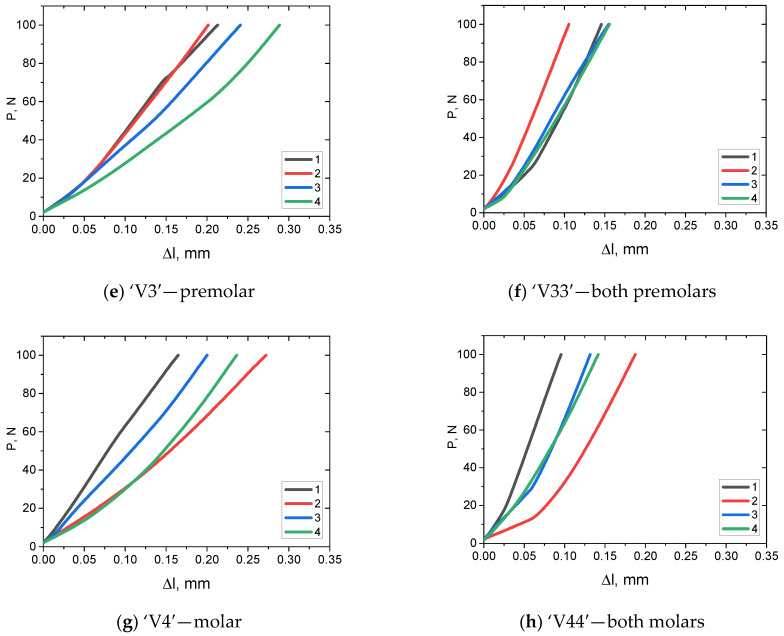
The ‘*P*–∆*l*’ load–displacement diagrams obtained in the mechanical tests: (1) ’HP+DT’ (**a**,**b**); (2) ‘3D+3D’ (**c**,**d**); (3) ‘3D+DT’ (**e**,**f**); and (4) ‘3D+CAM’ (**g**,**h**).

Figure 4 shows the ‘*P*–∆*l*’ load–displacement diagrams, according to which the mechanical properties of the studied RCDs were numerically assessed. Under application of the asymmetric point load on individual teeth (Figure 4a,c,e,g), the diagrams had a more linear pattern for all RCDs, while they exhibited an inclination angle, enhanced with rising grip displacement in the cases of symmetrical loading of the same teeth (Figure 4b,d,f,h). In addition, the ∆*l* values gradually decreased from the incisors to the molars when the maximum *P* load of 100 N was reached.

Despite the fact that the authors tried to reproduce the design of each type of the RCDs as closely as possible, the deformation response of the ‘tooth (pair of teeth)—denture base’ systems could vary for a number of reasons: (i) the difference in the properties of the cosmetic DT from those fabricated via 3D printing; (ii) the DT fastening in the 3D-printed base being ‘not ideal’ (both in terms of adhesion and ‘fit’); and (iii) the properties of the 3D-printed denture bases differing somewhat in their different parts, etc. In addition, since a lot of data were recorded for the RCDs, it was necessary to propose an approach to their multicriteria analysis. At the same time, it was also required to differentiate both the test results and the production routes.

### 3.2. Description of the Approach to the Interpretation of the Mechanical Test Data

In all mechanical tests under the point compressive load with a pin 3 mm in diameter to one or two denture teeth, the key condition was preset; namely, the tests were stopped when the load level of 100 N was reached (in addition to the reproducibility of the data of several successive records). This fact corresponded to the average statistical value accepted by most researchers in dentistry, which was characteristic of mastication [15,16]. The applied installation of dentition (both cosmetic denture teeth and ones made according to the digital protocols) primarily assumed that their strains were elastic. When testing the samples of the structural materials, this was described by Hooke’s law and was characterized by linear stress–strain relationships (for ones of a given shape and a known cross-section). For the RCDs, the components of which (the denture bases and the dentitions) could be made of different materials or possessed variable cross-sections, the pattern of the ‘*P*–∆*l*’ load–displacement diagrams was not linear in all cases. As mentioned above, the reasons for this phenomenon were the following:

- The contact between the loading pin with a diameter of 3 mm and the supporting ‘platform’ of a tooth (for example, the molar tubercle) was not absolutely plane-parallel, primarily due to the different shape of the surface of the teeth and the occlusion of the antagonist ones provided by this fact;
- The cosmetic denture teeth were fixed both in the base indentations and were connected to each other with the cured polymer, so they could adapt to the loading conditions, also due to their possible (even small) inclination relative to the applied load axis;
- The attachment conditions and the contact areas to the denture bases were different for various blocks of teeth (incisors, canines, premolars and molars);
- In the ‘3D+3D’ case, the properties of the polymers of the denture teeth (fitted and digitally produced) and the base, as well as the conditions of their attachment and pairing with neighbors (‘connection’ formed in 3D printing), could differ;
- Even a slight change in the tooth axis relative to the applied load direction could affect the shape and angle of the ‘*P*–∆*l*’ load–displacement diagrams.

Taking into account the mentioned features, the reaction was evaluated not for materials but for parts of the RCDs. Accordingly, their reaction could be non-linear, and it was necessary to decide how to interpret and apply the obtained experimental curves, as well as what quantitative parameters could be summarized from these data.

For example, Figure 4a shows the ‘*P*–∆*l*’ load–displacement diagram for the V1 scheme (one incisor, asymmetrical). As mentioned above, the analog protocol (curve 1) was taken as a reference. However, this fact did not mean that other production routes could not result in greater mechanical properties. Thus, curve 1 was of a non-linear type. Up to a ∆*l* displacement value of 0.15 mm, it was characterized by the maximum slope angle, while its growth began to deviate from the linear trend then. Being evaluated based on the maximum inclination angle (which, in fact, was proportional to the elastic modulus), it was characteristic only for the first loading stage. The subsequent deviation from the linear trend could indicate a decrease in the bearing capacity (of this part of the structure). Therefore, some questions were relevant. Firstly, how can we quantitatively characterize such a bearing capacity reduction? Secondly, since strains were assumed to be elastic, what was the correct way of interpreting its behavior in terms of mechanics? Thirdly, what quantitative metric could be applied in this case?

I. Since the curve behavior resembled irreversible strains with rising loads, it could be assumed that such a decrease could be interpreted as insufficient reliability of the RCD design. This suggestion enabled us to introduce reliability as the first characteristic, the quantitative measures (metrics) of which could be obtained via an analysis of the ‘*P*–∆*l*’ load–displacement diagrams if the curves began to change (decrease) non-linearly with rising loads.

II. Achieving the *P* load of 100 N corresponded to a certain ∆*l* displacement value. In this case, the same displacement could correspond to a load change of a different pattern, i.e., the curve rising trajectory. It was clear that the smaller the strain at this load, the less compliance the considered part of the RCD structure possessed. Nevertheless, it was proposed to use the ratio of the maximum load to the displacement level when it was reached as a stiffness criterion since the load–displacement diagrams characterized the properties of more than a single material. In contrast to the previous case (for reliability), the non-linearity degree of the curve behavior was not considered quantitatively. It should be noted that compliance was a measure of the inverse stiffness, which in turn was related to the elastic modulus.

III. Since shapes of the load–displacement diagrams could be convex or concave, this should also affect the performance of the RCDs. In particular, curve 4 in Figure 4a was characterized by maximum strains (in this case, plotted in units of the ∆*l* grip displacement) at low loads, while the growth rate increased as the external applied stress enhanced. In fact, curves 2 and 4 eventually ‘met’ at the same point. According to the previous stiffness criterion, the ‘3D+3D’ and ‘3D+CAM’ production routes provided identical properties of these RCDs. Since the RCDs were subjected to negligible loads in most cases, curve 4 could be classified in terms of fatigue as characterized by less durability (on the principle that greater displacements resulted in more severe damage accumulation). Once again, the issue of finding a metric for the quantitative characterization of durability by analyzing the load–displacement diagrams remained relevant.

Thus, it was proposed to quantitatively characterize the load–displacement diagrams of the studied RCDs according to the reliability, compliance/stiffness and durability criteria. The authors emphasized that all of them were rather conditional, both in terms of the way they were calculated and because the testing applied to not the materials but the (dental) structures. However, these statements enabled us to apply the criteria for the interpretation of the results of the mechanical tests.

## 4. Development of the Quality Assessment Criteria and Ranking of the Production Routes

The process of making a decision on the selection of the best production route for the RCDs according to the justified set of criteria and theoretical canons started with the identification of alternative solutions, which were the four options described above. As the factors characterizing each alternative, it was necessary to use the quantitative results of the mechanical tests under the point load applied at different points. Three criteria for assessing the quality of the RCDs were substantiated above: reliability, durability and compliance/stiffness, on the basis of which the quantitative values (factors) were determined.

The need to compare alternatives typically arises when there is a contradiction between the results of a comparison or the absence of an alternative that has the best performance of all factors. In this case, the problem of multicriteria optimization should be solved, namely, the selection of a rational alternative from an available finite set, i.e., an alternative that is closest to ‘ideal’. In addition, the multicriteria optimization tools make it possible to assess the degree of difference of all other alternatives from the rational one.

The AHP, TOPSIS, VIKOR, ELECTRE and PROMETHEE multicriteria optimization methods are among the most well-known ones [10,17,18]. Many published papers have been devoted to a comparison and evaluation of their capabilities [19,20,21]. Earlier, in an article by the authors [5], it was shown that it is sufficient to use the combined AHP-VIKOR method for solving the problems of the selection of dental materials. In this study, an algorithm was developed for ranking the mechanical properties. Correspondingly, the decision on the selection of the best production route for the RCDs was based on these data.

### 4.1. Metrics and Criteria for Evaluating the Production Routes for the RCDs

#### 4.1.1. The Reliability Criterion

It was assumed that a load–displacement diagram of an absolutely elastic material (structure) follows a linear law: $P\left(∆l\right)=P_{0}+\mu \cdot ∆l$. Any deviation $\varepsilon =P\left(∆l\right)-(P_{0}+\mu \cdot ∆l)$ from the linear law should lead to a loss of bearing capacity and, accordingly, the structure reliability. The following factors can be used as the basis for possible estimates of the deviation of the load–displacement diagram from the linear law:

- An assumption about the variability of the $P^{\prime }\left(∆l\right)$ derivative (range of values, standard deviation, etc.) [22,23,24];
- Statistical characteristics of the $P\left(∆l\right)$ approximation by a first-order polynomial due to the random pattern of the *ε* deviation (residual sum of squares of the RSS approximation, the *R*^2^ determination coefficient, etc.) [25,26];
- Integral geometric parameters of a graph (for example, the Ginny coefficient, the coefficient of non-linearity along an arc length) [27,28].

The above-described characteristics are discussed separately below. It is known that the estimate of the first derivative is an unstable characteristic, i.e., small deviations of the initial data lead to large deviations of the estimate itself. Therefore, it is necessary to apply preliminary smoothing of the data for calculating the estimate of the first derivative and use the obtained results then. For the studied case, smoothing in a sliding window (aperture) is the best option. By estimating the first derivative, it is possible to assess the non-linearity index in the form of a standard deviation of the derivative:

(1) $$Std=\sqrt{\frac{1}{N-1}\sum_{i=1}^{N}{\left(P^{\prime }\left({∆l}_{i}\right)-\bar{P^{\prime }}\right)}^{2}}$$

where *N* is the sample size and $\bar{P^{\prime }}$ is the derivative average value.

If a curve (a load–displacement diagram) is approximated by a first-order polynomial, i.e., represented in the form $f\left(∆l\right)=P_{0}+P_{1}∆l$, where $P_{0}$ and $P_{1}$ are the polynomial coefficients obtained via the least squares method, then the errors of such an approximation can be characterized by the residual sum of squares:

(2) $$RSS=\sum_{i=1}^{N}{\left(P\left({∆l}_{i}\right)-f\left({∆l}_{i}\right)\right)}^{2}$$

or by the determination coefficient:

(3) $$R^{2}=1-\frac{RSS}{\sum_{i=1}^{N}{\left(P\left({∆l}_{i}\right)-\bar{P}\right)}^{2}}$$

where $\bar{P}$ is the average value of the load–displacement diagram.

Among the geometric features of the difference between a load–displacement diagram and a linear graph, the area non-linearity coefficient should be mentioned, which is based on the Ginny coefficient [27]. It is determined as the ratio of the area bounded by a $P\left(∆l\right)$ graph and a *φ*(∆l) straight line drawn from a $P\left({∆l}_{1}\right)$ point to another $P\left({∆l}_{N}\right)$ one to the zone bounded by the $P\left(∆l\right)$ graph and axes. However, the Ginny coefficient, unlike the determination one, is interpreted in the opposite sense: the zero value indicates linearity, and equality to one reflects an absolutely non-linear law. Therefore, for the convenience of comparison, the inverse Ginny coefficient can be applied:

(4) $${NC}_{S}=1-\frac{\left|S_{D}-S_{∆}\right|}{S_{∆}}=1-\frac{\left|\sum_{i=2}^{N}\left(P\left({∆l}_{i}\right)+P\left({∆l}_{i-1}\right)\right)\left({∆l}_{i}-{∆l}_{i-1}\right)-\left(P\left({∆l}_{N}\right)+P\left({∆l}_{1}\right)\right)\left({∆l}_{N}-{∆l}_{1}\right)\right|}{\left(P\left({∆l}_{N}\right)+P\left({∆l}_{1}\right)\right)\left({∆l}_{N}-{∆l}_{1}\right)}$$

where $S_{D}$ is the area under the *P*(∆*l*) curve and $S_{∆}$ is the area under the *φ*(∆l) straight line.

By analogy with the coefficient of non-linearity along an arc length, given in [29], the ratio of the *φ*(∆l) straight line length to the *P*(∆l) curve length should be described:

(5) $${NC}_{L}=\frac{L_{\varphi }}{L_{P}}$$

where

$$L_{\varphi }=\sqrt{{\left({∆l}_{N}-{∆l}_{1}\right)}^{2}+{\left(P\left({∆l}_{N}\right)-P\left({∆l}_{1}\right)\right)}^{2}}$$

is the *φ*(∆l) straight line length on the $∆l\in \left[{∆l}_{1},{∆l}_{N}\right]$ and

$$L_{P}=\sum_{i=2}^{N}\sqrt{{\left({∆l}_{i}-{∆l}_{i-1}\right)}^{2}+{\left(P\left({∆l}_{i}\right)-P\left({∆l}_{i-1}\right)\right)}^{2}}$$

is the *P*(∆l) curve length on the $∆l\in \left[{∆l}_{1},{∆l}_{N}\right]$ segment.

Table A1 (Appendix A) presents the calculated values of the non-linearity coefficients according to the above expressions (1)–(5).

Obviously, all possible non-linearity estimates are mutually correlated, and it is enough to select one of them to solve a decision-making problem. The selection of the most informative of the correlated features is typically considered from the point of view of increasing the reliability or minimizing the losses of the final problem solution. Thus, it is carried out according to the training data set [30,31]. In the studied case, the estimates can be compared without training samples through the application of one of the well-known measures of informativeness. For example, in the Shannon method [32], entropy is used for this purpose:

(6) $$I\left(x\right)=-\sum_{k=1}^{q}P_{k}{log}_{2}P_{k}$$

where *q* is the number of the *x* magnitude gradations and $P_{k}$ is the probability of *x* falling into the *k*-th gradation. In the decision-making methods, data are normalized to the interval from 0 to 1. For the studied case, the experimental results should be ranked according to the four production routes, so the *q* number of gradations should also be taken as 4.

**Figure 5:**
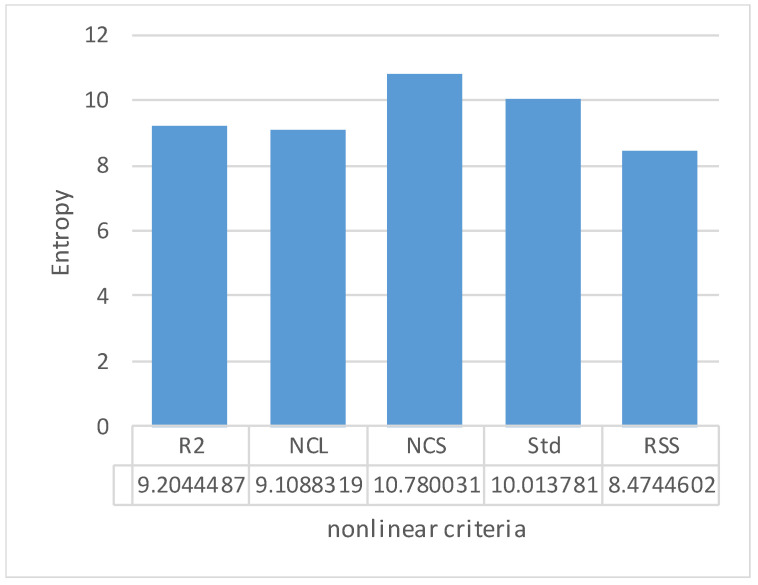
Comparison of the estimates and non-linearity coefficients.

According to Table A1 of Appendix A, the entropy of each non-linearity coefficient was calculated (Figure 5). According to the obtained results, all of the coefficients turned out to be close in information content, but the largest entropy value was noted for the geometric coefficient of non-linearity over the *NC*_S_ area. The advantages of this coefficient are that it is unlimited within the [0, 1] range and dimensionless. Consequently, the best quality of the RCDs manufactured by implementing the studied production routes was achieved when the *NC*_S_ coefficient tended to 1. Therefore, its maximization was considered the reliability criterion.

#### 4.1.2. The Durability Criterion

The second derivative characteristics of a graph, as indicators of its concavity (mean value, $P^{\prime \prime }\left(∆l\right)$ median), or the results of approximation by a second-order polynomial (the second-order variable coefficient) can serve as estimates of durability. These values are determined by using the following expressions:

1. The average value of the second-order derivative:
  (7) $$\bar{P_{2}}=\frac{1}{N}\sum_{i=1}^{N}P^{\prime \prime }\left({∆l}_{i}\right)$$
2. The median of the second-order derivative. If the $p_{i}=P^{\prime \prime }\left({∆l}_{i}\right)$ sequence of numbers is sorted in ascending or descending order, then the number located in the middle of this sequence is taken as the median value:
  (8) $$\bar{M_{2}}=p_{\left\|N/2\right\|}$$
3. When approximating a load–displacement diagram by the $f\left(∆l\right)=P_{0}+P_{1}∆l+P_{2}{∆l}^{2}$ second-order polynomial, the $P_{2}$ coefficient is determined via the least squares method by solving a system of linear algebraic equations:
  (9) $$\left\{\begin{array}{l} \left(\sum_{i=1}^{N}{∆l}_{i}^{4}\right)P_{2}+\left(\sum_{i=1}^{N}{∆l}_{i}^{3}\right)P_{1}+\left(\sum_{i=1}^{N}{∆l}_{i}^{2}\right)P_{0}=\sum_{i=1}^{N}{∆l}_{i}^{2}P\left({∆l}_{i}\right),\\ \left(\sum_{i=1}^{N}{∆l}_{i}^{3}\right)P_{2}+\left(\sum_{i=1}^{N}{∆l}_{i}^{2}\right)P_{1}+\left(\sum_{i=1}^{N}{∆l}_{i}\right)P_{0}=\sum_{i=1}^{N}{∆l}_{i}P\left({∆l}_{i}\right),\\ \left(\sum_{i=1}^{N}{∆l}_{i}^{2}\right)P_{2}+\left(\sum_{i=1}^{N}{∆l}_{i}\right)P_{1}+NP_{0}=\sum_{i=1}^{N}P\left({∆l}_{i}\right). \end{array}\right.$$

**Figure 6:**
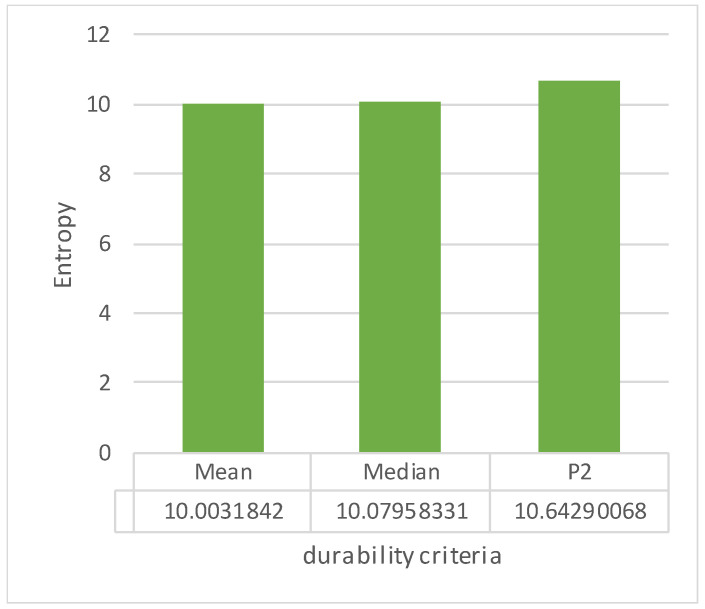
Comparison of the estimates and the durability criteria.

Table A2 of Appendix A presents such values calculated according to the experimental load–displacement diagrams. Similarly, based on the reasons described above, the informativity of the durability estimates was analyzed using the Shannon Formula (6). The calculation results are presented in Figure 6. As in the analysis of the non-linearity coefficients, all durability estimates turned out to be close in terms of their informativity, but the $P_{2}$ coefficient possessed the highest entropy value. Taking this level as the basis of the durability criterion, its dimension (N/mm^2^) and unboundedness should be noted.

An interpretation of the $P_{2}$ parameter enabled us to draw ambiguous conclusions. On the one hand, it was desirable that the load–displacement diagram was close to the linear law. In this case, the $P_{2}$ coefficient should tend to zero. On the other hand, out of all of the deviations from the linear law, it was better to select the one that corresponded to the negative values of the $P_{2}$ coefficient and, accordingly, the convexity (in other words, upward concavity) of the graph. Therefore, if the requirement to minimize the $P_{2}$ coefficient was accepted as the durability criterion, then it was necessary either to combine it with the reliability criterion or to recognize it as less significant.

#### 4.1.3. The Compliance/Stiffness Criterion

Since only the ∆*l* displacement value at the *P* load of 100 N was considered the basis of the compliance/stiffness criterion, and the non-linearity of the load–displacement diagram was not taken into account, it was senseless to introduce any additional metrics. For this reason, only the ∆*l* displacement was estimated according to the obtained experimental data given in Table 2. The ∆*l* values (in millimeters) were limited on the left by zero. The compliance/stiffness criterion was based on the ‘cost’ principle (the smaller the ∆*l*, the better).

### 4.2. Results of Applying the Combined AHP-VIKOR Method

In ranking the production routes for the RCDs, the first step was to determine the weighting coefficients of the applied quality criteria. According to the analytic hierarchy process (AHP) method, the weights of the criteria were calculated using a pairwise comparison table [33]. Such a table was formed by experts, so it was subjective. Therefore, either fuzzy set methods had to be used [34], or it was necessary to analyze the difference in the expert opinions to obtain a more objective assessment. In this study, the authors used the latter approach and calculated the weights using alternative tables.

The following scale was implemented to assess the pairwise significance:

1—The criteria were equivalent;

3—The first criterion had a slightly greater significance than the second one;

5—The first criterion was characterized by a substantially greater significance than the second one;

7—The first criterion was undeniably more important than the second one, since this fact was confirmed by the experts and since it was standard practice as well;

9—The first criterion possessed absolutely greater significance than the second one.

The cells of the pairwise comparison table with symmetric indices were inversely related $a_{i,j}=\frac{1}{a_{j,i}}$. For example, if the *i*-th criterion had a much greater significance than the *j*-th one, then $a_{i,j}=5$ and $a_{j,i}=\frac{1}{5}$. The criteria weights were calculated by searching for the eigenvalues of a matrix formed in this way.

The second stage of ranking was based on the VIKOR method, i.e., based on the calculation of three metrics for normalized functions [21]:

(1) The weighted Manhattan distance to an ideal alternative consisting of the ‘best’ factor values:

(10) $$S_{i}=\sum_{j=1}^{n}w_{j}\left[\frac{f_{j}^{*}-f_{i,j}}{f_{j}^{*}-f_{j}^{-}}\right]$$

(2) The weighted Chebyshev distance:

(11) $$R_{i}=\underset{j}{\max}w_{j}\left[\frac{f_{j}^{*}-f_{i,j}}{f_{j}^{*}-f_{j}^{-}}\right]$$

where $f_{i,j}$ was the *j*-th criterion value for the *i*-th alternative; $f_{j}^{*}$ was the best value of the *j*-th criterion among all alternatives; $f_{j}^{-}$ was the worst value of the *j*-th criterion among all alternatives; and *w_j_* was the *j*-th criterion weight.

(3) The intermediate value of the above metrics, otherwise rational:

(12) $$Q_{i}=v\frac{S_{i}-S^{*}}{S^{-}-S^{*}}+(1-v)\frac{R_{i}-R^{*}}{R^{-}-R^{*}}$$

where $S^{*}=\underset{i}{\min}S_{i}$, $S^{-}=\underset{i}{\max}S_{i}$, $R^{*}=\underset{i}{\min}R_{i}$, $R^{-}=\underset{i}{\max}R_{i}$, and v was the weight of the strategy of the ‘majority of criteria’.

The values (10)–(12) were limited within the [0, 1] range, and they could be interpreted as the pessimistic, optimistic and rational assessments of the alternative position in the set, respectively. The ‘0′ value meant that the alternative achieved the ‘best’ quality according to all criteria, while the ‘1′ level was the ‘worst’ of the available ones. The alternatives were ranked as follows. Firstly, the $Q_{i}$ rational estimate was ordered. Then, the difference between the nearest ordered $Q_{i}$ values was compared with the 1(m−1) parameter, where m was the number of alternatives. Finally, a decision was made about the equality of their ranks.

By applying the described criteria, the production routes for the RCDs were ranked according to their individual parts initially. Then, the loading points were taken into account as well, which were obviously more related to the operation (failure/fracture) statistics of such products.

#### 4.2.1. Ranking the Production Routes for the RCDs by Their Individual Parts

Tables of pairwise comparisons of the criteria were filled in according to two principles: the equivalence of the criteria and the reliability preference. The equivalence of the criteria led to the equality of their weights. According to the reliability preference, the results of pairwise comparisons of the criteria and their weights are presented in Table 3.

**Figure 7:**
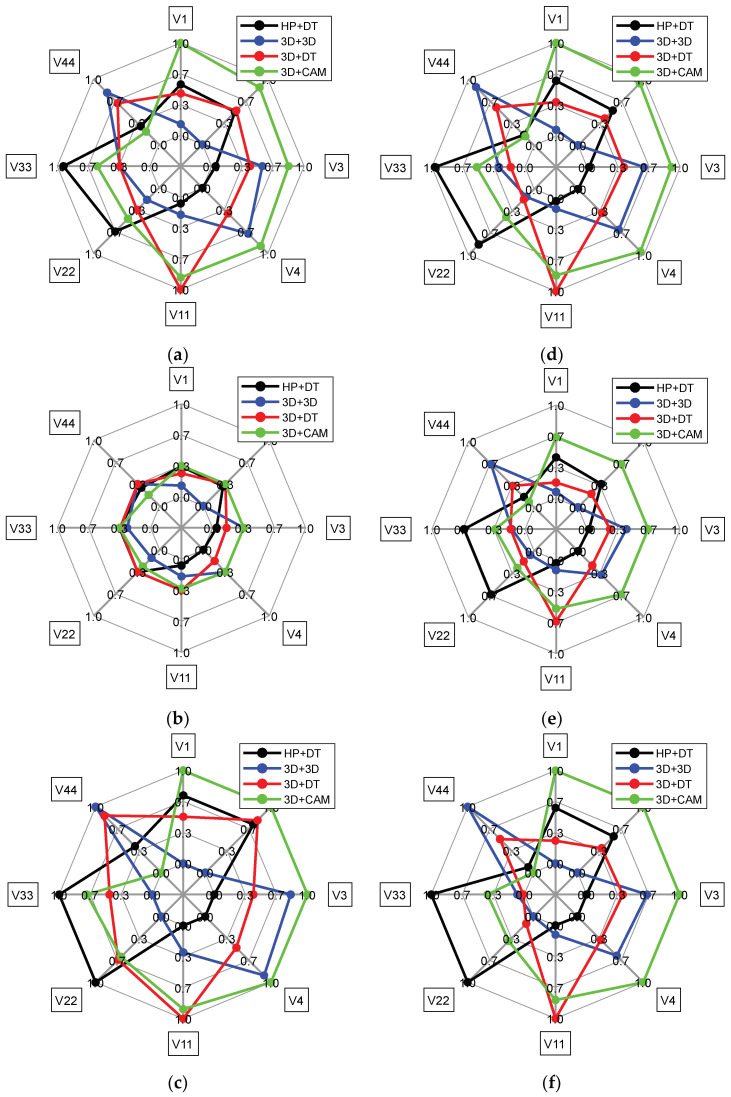
Radial diagrams of the *S* (**a**,**d**), *R* (**b**,**e**) and *Q* (**c**,**f**) estimates at the loading points of the RCDs for the cases of the equivalence of the criteria (**a**–**c**) and the reliability preference (**d**–**f**).

For the case of the equivalence of the criteria, the calculated metrics according to the characteristics of the loading points are given in Table 4 and Figure 7a–c. These data showed that none of the alternatives had an absolute advantage since each of them possessed at least one loading point with the best mechanical characteristics. The optimistic *R* assessment enabled us to conclude that all alternatives could have ‘good’ properties (values from 0 up to 0.3) for all loading points. Based on the number of loading points with the achievement of the rational properties, the ‘3D+3D’ production route stood out (the first five ranks), while the smallest number was noted for the ‘3D+DT’ one.

The metrics calculated from the characteristics of the loading points with the reliability preference are given in Table 5 and Figure 7d–f. These data also did not reveal the absolute advantage of any alternative. Both *R* optimistic and *S* pessimistic estimates showed great dispersions of the values for the loading points. However, in terms of their number with the achievement of the rational properties, the ‘3D+3D’ production route (the first five ranks) also stood out, while the ‘3D+CAM’ one had the smallest number of the first five ranks.

#### 4.2.2. Ranking of the Production Routes for the RCDs Considering the Loading Points (the Contribution of All Blocks of Teeth)

When filling the tables of pairwise comparisons of the loading points, three strategies were considered:

(i) The strategy of equal probability of the load application at the studied points assumed the equivalence of taking into account the contribution of all loading points (the pair comparison table, obviously, contained only units, while the weights were similar);

(ii) The ‘bite off’ strategy reflected that mainly the anterior blocks of teeth (incisors and canines) were loaded (Table 6).

(iii) The ‘mastication’ strategy suggested that posterior teeth (premolars and molars) were mainly used, both individually and as symmetrical pairs (Table 7).

For all loading modes, the ranking of the production routes for the RCDs was carried out by summarizing the results of pairwise comparisons of the criteria in Table 8.

According to the strategy of equal probability of the load application at the studied points, the optimistic *R* estimate gave equivalent values to all alternatives, which indicated the acceptability of all considered production routes. Both pessimistic *S* and rational *Q* assessments identified the ‘HP+DT’ and ‘3D+3D’ production routes as leaders, while the ‘3D+CAM’ one was recognized as an outsider (the worst of those considered).

The ‘bite off’ strategy with the reliability preference principle revealed the ‘3D+3D’ production route as an undisputed leader. The ‘HP+DT’ and ‘3D+DT’ ones were close in terms of their ratings and were characterized by average performance (quality) between the rational and worst production routes.

The ‘mastication’ strategy and the reliability preference principle highlighted another pair of rational production routes, namely, the ‘HP+DT’ and ‘3D+DT’ ones.

As a preliminary discussion, the authors put forward the following thesis. When loading on the anterior blocks of teeth (V1 and V2), the best results were shown by the ‘3D+3D’ RCD, while the ‘HP+DT’ and ‘3D+DT’ ones were favorable if the load was applied on the posterior blocks of teeth. This fact did not mean that in order to provide higher mechanical properties (deformation behavior), various parts of the RCDs had to be made via different production routes. Nevertheless, it could indicate that the design of the posterior half of the RCDs (blocks of both premolars and molars) should be corrected/optimized.

## 5. Computer Simulation of Loading the RCDs

Since the authors did not pay attention to aspects of the structural characterization of the RCDs manufactured via various production routes in this study, computer simulation methods were utilized to identify possible causes of the non-linear response of the dental structures [35,36,37]. As two varieties (deviations) of the load–displacement diagrams from elasticity, it was proposed to consider the following:

(1) The gradual ‘loss of bearing capacity’ (‘deviation’ of a curve to the right with a decrease in the tangent inclination angle, for example, curve 1 in Figure 4a);

(2) The gradual ‘deviation’ of a curve to the left (for example, curve 4 in Figure 4a), which reflects the ‘restoration’ of stiffness.

This behavior could be caused by both differences in the production routes of the RCDs (materials and procedures) and the presence of various discontinuities. However, such a simplified consideration was quite legitimate, since the purpose of this section was not to fully reproduce the experimental conditions but to simulate the behavior of a dental structure.

**Figure 8:**
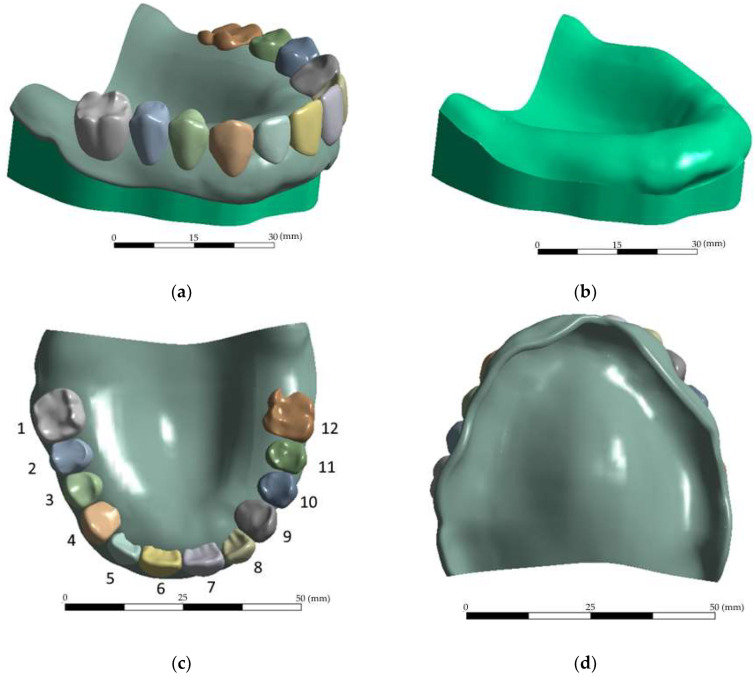
The RCD solid model (maxillary prosthesis) used in the calculations (**a**); the assembly model of the prosthesis on the support (**b**); and the top (**c**) and bottom (**d**) views of the prosthesis.

A model of the maxillary RCD, implemented for the FEM-based computer simulation, is shown in Figure 8a. It was a dentition consisting of twelve PMMA teeth mounted on a PMMA base. The teeth imitated denture teeth in their structure, so they were not connected to each other, while the average distance between them was ~100 µm. Similar to the experimental examinations, the RCD was mounted on a steel support before loading (Figure 8b). The principle of free fixation of the inner part of the denture base on the upper surface of the steel support was implemented. The contours of the mating surfaces geometrically fitted each other exactly (Figure 8d), which excluded the formation of gaps. The laboratory experiment conditions were physically simulated when the required load was applied to the individual teeth (Figure 8c), while the lower surface of the support was rigidly fixed. The boundary conditions between the teeth and the denture base were set as a rigid contact, excluding slippage and movement. In Figure 8c, the numbers of the teeth are marked as in the reported results: 1, 12—molars; 2, 3, 10, 11—premolars; 4, 9—canines; and 5, 6, 7, 8—incisors. The problem was solved by using the FEM in a linear elastic formulation and the Lagrangian implementation. As the properties of isotropic materials in the calculations, the following values were taken: the elastic modulus of 1/2/210 GPa and the Poisson’s ratio of 0.3/0.3/0.33 for the denture base/dentition/metal base, respectively.

**Figure 9:**
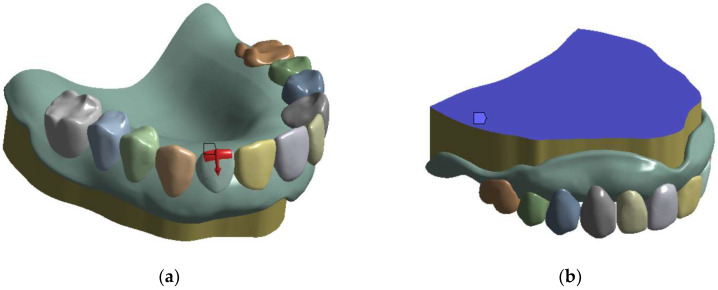
The loading boundary conditions for the incisor (tooth No. 5) at the load of 50 N (**a**); the kinematic boundary conditions of the rigid fixation of the support base (**b**).

When constructing the model, its boundary conditions were varied, the contact pairs were adjusted, and the solver settings were selected. In this case, an axial load of 50 N (shown by the arrow in Figure 9a) on the second right incisor (tooth No. 5 in Figure 7c) was taken as the ‘intermediate’ boundary condition. The kinematic boundary conditions were considered the rigid fixation of the support base (the surface indicated by the arrow in Figure 9b).

### 5.1. The Model Testing

**Figure 10:**
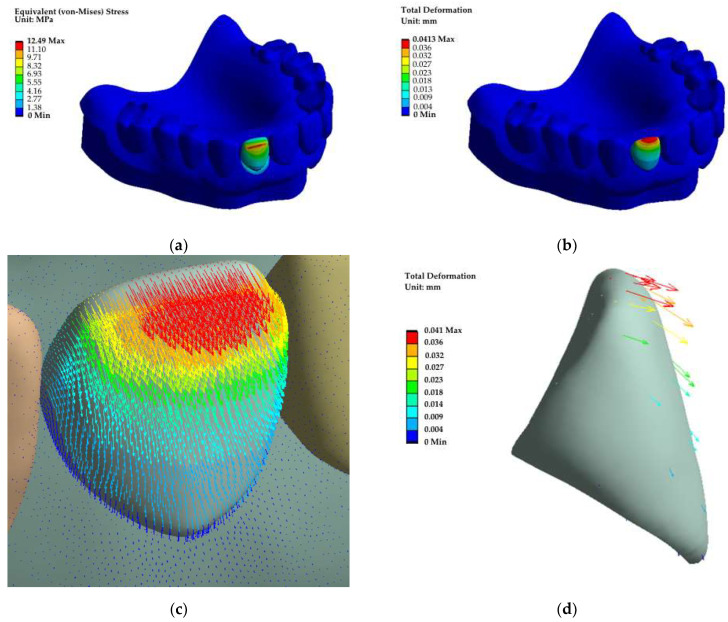
The fields of equivalent stresses (**a**) and total displacements (**b**) in the loaded RCD, as well as vector representations of the displacements in the model (**c**) and the tooth (**d**).

As an intermediate (testing) result, the distribution fields of the components of the stress tensor, strains and the displacement vector are shown. The von Mises distributions of equivalent stress fields (within the framework of the corresponding strength theory) are presented in Figure 10a. According to these data, the *σ*_max_ maximum stress level of 12.5 MPa was concentrated in the subsurface layer of the upper part of the loaded tooth. In this case, the maximum total ∆*l*_max_ displacement was 41 µm (Figure 10b).

To visualize the effect of an axial load on the displacement redistribution in the dental structure, the total values were decomposed into individual components. From the vector representation shown in Figure 10c,d, it could be concluded that a bending moment emerged despite the load application along the tooth axis due to the complex shape and the curvilinear pattern of the connection with the denture base. It contributed to the displacement of the upper part of the tooth at a significant angle to the applied load direction.

Thus, it was shown at the testing stage that the application of a compressive load along the tooth axis could result in a non-linear response of the structure, including the development of overturning forces.

### 5.2. The Results of the FEM-Based Computer Simulation

#### 5.2.1. The First Block (Incisors, Teeth No. 6 and 7, Fz = 100 N; No. 6–7, Fz = 50 + 50 = 100 N)

**Figure 11:**
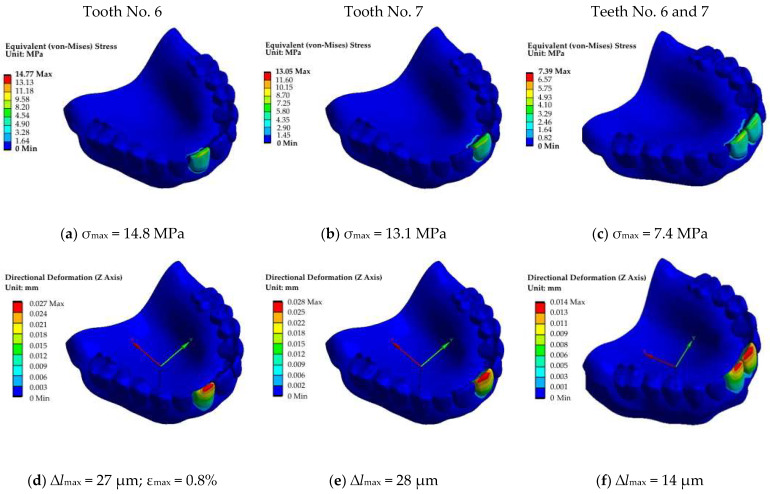
The fields of equivalent stresses (**a**–**c**) and displacements (blue axis) (**d**–**f**) under the application of the load on the incisors: No. 6 (**a**,**d**), No. 7 (**b**,**e**) and No. 6 and 7 (**c**–**f**).

**Figure 12:**
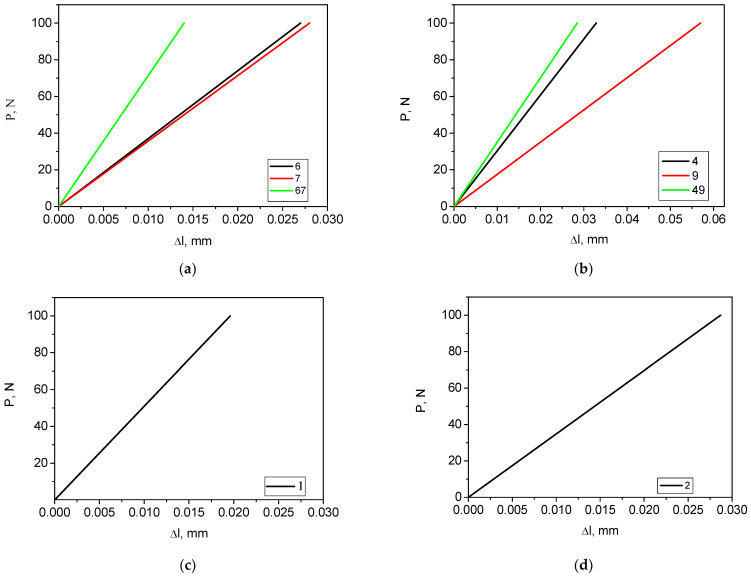
The load–displacement diagrams for the uniaxial compression scheme. The load applied to teeth No. 6 and 7 as well as both No. 6 and 7 (**a**); No. 4 and 9 as well as 4–9 (**b**); No. 1 (**c**); and No. 2 (**d**).

At the first simulation stage, the load was applied to the first block of teeth, i.e., incisors (Figure 11). When the teeth were loaded one at a time, their *σ*_max_ maximum levels of 13.1–14.8 MPa were comparable, while they decreased by approximately a factor of two under symmetrical loading (Figure 11a–c), as expected. A similar pattern was also typical for the distribution of displacements (Figure 11d–f). The corresponding load–displacement diagram, shown in Figure 12a, was in good agreement with the fields of equivalent stresses and displacements. It could also be stated that the calculated (Figure 11a) and experimental (Figure 4a,b) diagrams were characterized by quantitative agreement. This fact additionally indicated that the RCD model correlated well with the experimental samples.

#### 5.2.2. The Second Block (Canines, Teeth No. 4 and 9, Fz = 100 N; No. 4–9, Fz = 50 + 50 = 100 N)

**Figure 13:**
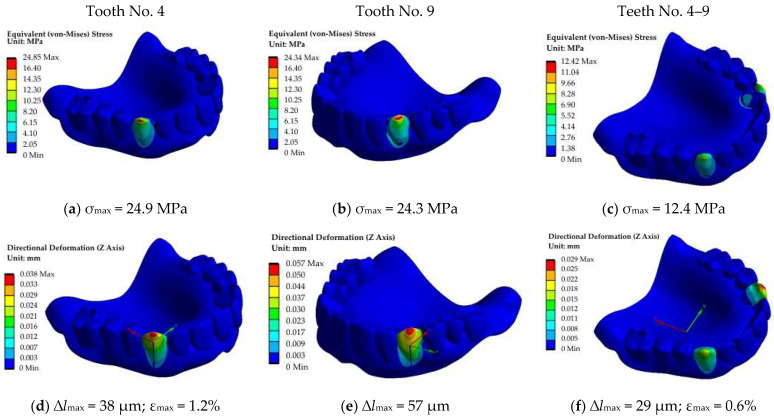
The fields of equivalent stresses (**a**–**c**) and displacements (blue axis) (**d**–**f**) under the application of the load on the canines: No. 4 (**a**,**d**), No. 9 (**b**,**e**) and No. 4–9 (**c**–**f**).

At this simulation stage, the load was applied to the second block of teeth (Figure 13). The pattern of the stress distribution was generally preserved, although their values increased by ~10 MPa (Figure 13a–c). However, such a trend was not typical for the displacement field (Figure 13d–f). When loading tooth No. 9, the displacement value almost doubled. This phenomenon can be clearly observed in the corresponding diagram shown in Figure 12b. According to the authors, the obtained result was related to the specifics of the implemented structure of the RCD model. This indicated that the experimentally observed variations of the load–displacement diagrams for the studied RCDs could be caused not by the presence of heterogeneities/discontinuities (for example, in 3D printing) but by some features of a particular part of the structures.

#### 5.2.3. The Third and Fourth Blocks (Molar and Premolar, Teeth No. 1 and 2, Fz = 100 N)

**Figure 14:**
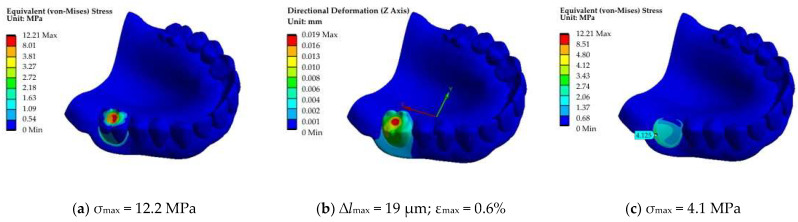

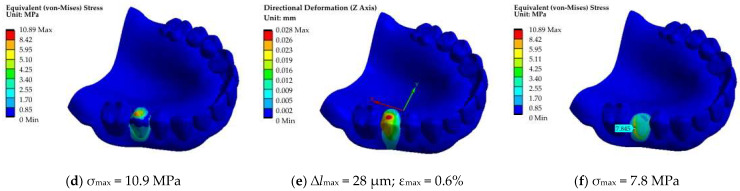
The fields of equivalent stresses (**a**,**c**,**d**,**e**) and displacements (blue axis) (**b**,**d**) under the application of the point load on the molar No. 1 (**a**,**b**,**c**) and the premolar No. 2 (**b**,**e**); the loaded tooth is not shown in (**a**,**c**,**d**,**f**).

Because the molar is characterized by a large flat area on the top, a circular shape zone with a diameter of 3 mm was allocated for loading. It was assumed that the indenter (pin) with a diameter of 3 mm, fixed in the upper plate of the grip, pressed on this platform. Thus, the load was applied to one tooth only (Figure 14). Additionally, equivalent stress fields without the loaded tooth are shown in Figure 14c,f), while the corresponding load–displacement diagrams are presented in Figure 12b,c.

In the case of the load application to the molar, the displacements were minimal (Figure 14a–c). This result was related to the maximum area of its support. The *σ*_max_ maximum stresses of 10.9–12.2 MPa were comparable.

Before discussing the obtained results, it should be emphasized that no full agreements between the laboratory and numerical experiments could be expected, since the RCD structure was not studied in detail and was not reproduced accurately. The authors tried to make a rather qualitative comparison and, among other things, to trace the pattern of the distributions of both stresses and displacements (when performing the calculations in the elastic statement).

## 6. Discussion

The development of digital dentistry is an ultimate trend nowadays. For this reason, most of the recent papers on the development of denture bases are dedicated to aspects of 3D printing [38,39]. Thus, it is expected that comparisons are continuously conducted between conventional PMMA and 3D-printed resins for denture bases [40,41]. Some of the criteria for comparing the properties of dentures, fabricated by using subtractive and additive manufacturing technologies, besides the key strength ones [42,43], are color stability [44], processing deformations [45], dimensional stability [46], resistance to immersion in different coloring agents [47], etc.

The results of mechanical testing illustrated in the paper agree well in the qualitative and quantitative sense with those presented elsewhere in [48,49]. In doing so, the 3D-printing equipment, feedstocks and post-build treatment modes could vary substantially. The particular feature of the current study is related to the localized (point) pattern of mechanical loading applications to single (asymmetrical) or couple (symmetrical) artificial teeth with the rigid fixation of the denture base on the metal foundation.

Although the study included three parts, the general purpose was to substantiate the applicability of the digital protocol for manufacturing RCDs with an acceptable response to external loads. The variety of the obtained results necessitated their ranking. However, such data processing could and should be carried out by not just taking into account both full-scale and computational experiments. No less important are biological, economic and technological indicators [5]. The obtained results indicated that 3D printing is not only acceptable but can be a priority production route as well. At the same time, the RCD cost indicators can be significantly reduced in their mass production.

It should be noted that the following assumption was applied for the ‘3D+DT’ production route. The probability of the presence of discontinuities in denture teeth was minimal. Therefore, the decrease in the mechanical properties (in terms of reliability, durability and compliance/stiffness) was determined mainly based on the ‘shortcomings’ of the 3D-printed denture base. The ‘3D+CAM’ RCD had to possess properties similar to those of the ‘3D+DT’, since the blocks from which the CAD/CAM teeth were milled were also factory-fabricated and their structure could not deteriorate in the turning process. Thus, the difference was mainly caused by the properties of the denture base material (or the conditions for fixing the teeth in the denture base’s indentations). 

In three cases (V1, V3 and V4) out of eight, the compliance of the ‘3D+DT’ RCD was lower, and its curve was quite similar to those of ‘3D+DT’ and ‘3D+CAM’ in all symmetrical loading cases. Thus, the four studied RCDs could be roughly divided into three subclasses, namely, the fully analog ‘HP+DT’, fully additive ‘3D+3D’ and partially additive ‘3D+DT’ and ‘3D+CAM’. According to the ranking results, it could be stated that the full 3D-printing production route proved the potential and competitiveness of the implementation of AM for such purposes.

The authors considered returning once again to the used durability and reliability concepts. In terms of the application of cyclic loads, durability is estimated according to the data from laboratory tests [50], which were not carried out in this study. Reliability is an even more complex parameter, which is based on an analysis of failure statistics. Note that the problem was not solved in this formulation for the reported cases. As such, an assessment of both durability and reliability may be the subject of further research. Since the authors obtained only the results of the static tests of the RCDs, these data were used to rank the production route significances.

The results of the FEM-based computer simulations showed that the load–displacement diagrams of the RCDs were linear, as expected. In fact, the causes of non-linearity in the loading experiment using the numerical methods were not clearly revealed. It might be solved by moving the prosthesis relative to the support base in the laboratory experiments. On the other hand, if the task was to experimentally reproduce the obtained load–displacement diagrams, then it was required to explicitly set both the presence and the properties of a transition layer in the model (which was used for fastening the teeth in the denture base). Thus, the linear-elastic model of the RCDs is to be replaced by a non-linear one (hyperelastic, for instance). In this case, a non-linear response of the entire RCD structure could be expected.

As a prospect, the authors propose to explicitly take into account the RCD structure and the specifics of the production routes. Based on many years of clinical experience in dental treatment associated with the installation of RCDs, an important criterion for their quality is, among other things, maintaining fixation in the process of biting off and mastication (as noted in Section 4 when substantiating the appropriate strategies). In contrast to the conditions implemented in this research, the possibility of RCD retention can be changed even during a single meal. As such, it is necessary to consider its resistance to overturning at the design stage. Partially, these conditions were verified in the mechanical tests. Thus, the ‘3D+3D’ production route showed RCD resistance to such applied loads.

The above results exhibit just a first step toward the development of an experimental–theoretic approach to studying the deformation behavior of RCD fabricated via 3D printing. Due to space limitations, the mechanical testing data of mandible RCD were left out of the paper. In addition, the only digital model was studied, but variations of its types as well as geometrical and constructional features would affect the obtained results. Of particular interest is deformation behavior under inelastic deformation and cyclic loads (fatigue) as well. Finally, the FEA should be conducted when the stress–strain state is estimated over the elastic statement. However, this does not deprive the obtained results of originality and actuality. 

It should be noted that the digital model was developed by taking the anatomical features of a particular patient. This 3D-printed maxillary RCD is in service these days. Thus, the clinical application of the developed AM prosthesis has been successfully proven. In addition, the proposed idea of an experimental–theoretical approach to the design of RCD is currently being introduced into the practice of dental treatment at the 3rd State Medical University (Moscow, RF).

## 7. Conclusions

Full-scale mechanical tests of the four RCDs, fabricated via AM (partial or full), were carried out. They were loaded both symmetrically and asymmetrically on each of the four blocks of teeth (incisors, canines, premolars and molars). It was shown that the response of the dental structures (the dentitions fixed on the PMMA bases) was characterized by the non-linear elastic stage in most cases under loads up to 100 N. At the same time, the test results did not allow us to classify any implemented production route as unsatisfactory.

To solve the ranking problem, it was proposed to interpret the results of the mechanical tests in terms of the reliability, durability and compliance/stiffness criteria. From the set of possible characteristics, the most informative ones were chosen as ranking factors, namely, the inverse Ginny coefficient of the load–displacement diagram, the *P*_2_ coefficient of the second-order approximation and the ∆*l* displacement value at a *P* load of 100 N. The production routes of the RCDs were ranked using the combined AHP-VIKOR method.

In the first stage, the loading points were ranked, i.e., the weights of the criteria were calculated according to the table of their pairwise comparisons. Both the calculated optimistic *R* and pessimistic *S* estimates showed a large spread of values at the loading points. However, according to the number where the rational properties were achieved, the ‘3D+3D’ production route took the first five ranks, while the ‘3D+CAM’ one possessed the smallest number of the first five ranks.

In the second stage, the production routes were ranked, taking into account the RCD loading mode according to three strategies: (i) the equal probability of the load application at the loading points; (ii) the ‘bite off’ strategy; and (iii) the ‘mastication’ strategy. With the first strategy, the optimistic *R* assessment gave equivalent values to all alternatives, which indicated the acceptability of all considered production routes. The pessimistic *S* and rational *Q* estimates identified two ‘HP+DT’ and ‘3D+3D’ production routes as the leaders. The ‘bite off’ strategy together with the principle of the reliability preference revealed the ‘3D+3D’ production route as the undisputed favorite. The ‘mastication’ strategy together with the principle of the reliability preference reflected another pair of rational production routes, namely, the ‘HP+DT’ and ‘3D+DT’ one.

The FEM-based computer simulation of the deformation response of the RCD model, representing a set of isolated PMMA teeth installed on a PMMA basis, was carried out under the load application in both symmetrical and asymmetric manners. Despite the fact that the numerical experiment was performed in the elastic formulation, a quantitative agreement of the results was obtained. The implemented model enabled us to identify the possible reasons for the difference in the pattern of the deformation behavior, in particular on an example of the loaded canines. The development of the model assumed the explicit consideration of both the structure and possible discontinuities in RCDs fabricated via the implemented production routes.

The significance of the study is related to the key conclusion that the use of the ‘3D+3D’ production route is a promising and cost-effective technology for manufacturing RCDs. This digital technique for customized prosthetic treatment can reduce the financial costs for patients, as well as the time and labor costs for doctors. The obtained results have confirmed that the mechanical behavior of the RCDs manufactured via the ‘3D+3D’ production route is not inferior to that found using the reference method, namely, the analog protocol. Further research development in this direction will be associated with an improvement of the mechanical and tribological properties of dentition via their AM from (glass) filled composites.

## Figures and Tables

**Table 1:** The production routes for manufacturing the RCDs.

No.	Production Route	Designation
1	‘Analog protocol’ (reference point) Hot polymerization (HP) of the denture base + cosmetic denture teeth (DT)	‘HP+DT’
2	(3D) DLP of both denture base and dentition	‘3D+3D’
3	(3D) DLP of the denture base + cosmetic denture teeth (DT)	‘3D+DT’
4	(3D) DLP of the denture base + CAD/CAM milled teeth	‘3D+CAM’

Reference data for PMMA: Shore D hardness ~58; density ~1190 kg/m^3^ [11].

**Table 2:** Displacement in the maxillary RCD at the load of 100 N.

No.	Production Route	Loading Point							
		V1	V11	V2	V22	V3	V33	V4	V44
1	‘HP+DT’	0.320	0.220	0.217	0.142	0.213	0.146	0.165	0.096
2	‘3D+3D’	0.263	0.202	0.212	0.159	0.202	0.106	0.272	0.187
3	‘3D+DT’	0.283	0.289	0.297	0.187	0.241	0.155	0.200	0.132
4	‘3D+CAM’	0.321	0.285	0.265	0.177	0.289	0.156	0.235	0.142

**Table 3:** The results of pairwise comparisons of the criteria and their weights according to the reliability preference.

	Reliability	Durability	Compliance/Stiffness	Weight
Reliability	1	5	3	0.66
Durability	1/5	1	1	0.16
Compliance/stiffness	1/3	1	1	0.19

**Table 4:** The metrics and the ranking results in the case of the equivalence of the criteria.

No.	Production Route	V1	V2	V3	V4	V11	V22	V33	V44
		*S*							
1	‘HP+DT’	0.551	0.4947	0.0442	0	0.0698	0.6667	0.933	0.2775
2	‘3D+3D’	0.1203	0	0.5487	0.7004	0.1918	0.1811	0.3219	0.7908
3	‘3D+DT’	0.4542	0.515	0.4151	0.3895	1	0.3333	0.3261	0.6299
4	‘3D+CAM’	1	0.8711	0.8314	0.8884	0.8702	0.4721	0.5688	0.1952
		*R*							
1	‘HP+DT’	0.3278	0.2983	0.0442	0	0.0698	0.3333	0.3333	0.2775
2	‘3D+3D’	0.1203	0	0.3333	0.3333	0.1865	0.1232	0.2511	0.3333
3	‘3D+DT’	0.2538	0.3333	0.1504	0.1696	0.3333	0.3333	0.3261	0.3333
4	‘3D+CAM’	0.3333	0.3333	0.3333	0.3333	0.3165	0.2548	0.3333	0.1669
		*Q*							
1	‘HP+DT’	0.7319	0.7313	0	0	0	1	1	0.4013
2	‘3D+3D’	0	0	0.8205	0.8942	0.2871	0	0	1
3	‘3D+DT’	0.5031	0.7956	0.4193	0.4737	1	0.6568	0.4592	0.8649
4	‘3D+CAM’	1	1	1	1	0.8982	0.6127	0.702	0
		Rank							
1	‘HP+DT’	3	2	1	1	1	4	4	1
2	‘3D+3D’	1	1	3	3	1	1	1	4
3	‘3D+DT’	2	3	2	2	4	3	1	3
4	‘3D+CAM’	4	4	4	4	3	1	3	1

**Table 5:** The metrics and the ranking results in the case of the reliability preference.

No.	Production Route	V1	V2	V3	V4	V11	V22	V33	V44
		*S*							
1	‘HP+DT’	0.5941	0.5277	0.0209	0.0000	0.0330	0.8422	0.9683	0.1555
2	‘3D+3D’	0.0674	0.0000	0.6103	0.6181	0.1149	0.1174	0.2799	0.8828
3	‘3D+DT’	0.3644	0.4049	0.3955	0.3636	1.0000	0.1578	0.1543	0.5750
4	‘3D+CAM’	1.0000	0.9390	0.9055	0.9472	0.8298	0.4249	0.5180	0.1345
		*R*							
1	‘HP+DT’	0.4389	0.3525	0.0209	0.0000	0.0330	0.6555	0.6555	0.1555
2	‘3D+3D’	0.0674	0.0000	0.4236	0.3561	0.1045	0.0583	0.1407	0.6555
3	‘3D+DT’	0.1678	0.2032	0.2462	0.2167	0.6555	0.1578	0.1543	0.3264
4	‘3D+CAM’	0.6555	0.6555	0.6555	0.6555	0.5171	0.2553	0.3193	0.0790
		*Q*							
1	‘HP+DT’	0.5982	0.5499	0.0000	0.0000	0.0000	1.0000	1.0000	0.0803
2	‘3D+3D’	0.0000	0.0000	0.6504	0.5979	0.0997	0.0000	0.0771	1.0000
3	‘3D+DT’	0.2445	0.3706	0.3892	0.3572	1.0000	0.1111	0.0132	0.5089
4	‘3D+CAM’	1.0000	1.0000	1.0000	1.0000	0.8008	0.3770	0.3969	0.0000
		Rank							
1	‘HP+DT’	3	3	1	1	1	4	4	1
2	‘3D+3D’	1	1	3	3	1	1	1	4
3	‘3D+DT’	1	2	2	2	4	1	1	3
4	‘3D+CAM’	4	4	4	4	3	3	3	1

**Table 6:** The results of pairwise comparisons of the loading points according to the ‘bite off’ strategy.

	V1	V2	V3	V4	V11	V22	V33	V44	Weight
V1 incisor	1	1	5	7	1	1	9	9	0.22
V2 canine	1	1	5	7	1	1	9	9	0.22
V3 premolar	1/5	1/5	1	1	1/5	1/5	1	1	0.04
V4 molar	1/7	1/7	1	1	1/7	1/7	1	1	0.03
V11 both incisors	1	1	5	7	1	1	9	9	0.22
V22 both canines	1	1	5	7	1	1	9	9	0.22
V33 both premolars	1/9	1/9	1	1	1/9	1/9	1	1	0.03
V44 both molars	1/9	1/9	1	1	1/9	1/9	1	1	0.03

**Table 7:** The results of pairwise comparison of the loading points according to the ‘mastication’ strategy.

	V1	V2	V3	V4	V11	V22	V33	V44	Weight
V1 incisor	1	1	1/9	1/9	1	1	1/7	1/7	0.03
V2 canine	1	1	1/7	1/7	1	1	1/7	1/7	0.03
V3 premolar	9	7	1	1	9	7	5	5	0.30
V4 molar	9	7	1	1	9	7	5	5	0.30
V11 both incisors	1	1	1/9	1/7	1	1	1/7	1/7	0.03
V22 both canines	1	1	1/9	1/7	1	1	1/7	1/7	0.03
V33 both premolars	7	7	1/5	1/5	7	7	1	1	0.14
V44 both molars	7	7	1/5	1/5	7	7	1	1	0.14

**Table 8:** The summarized ranking results for all studied strategies.

No.	Production Route	S	R	Q	Rank	Order
Equal probability strategy (v = 1)						
1	‘HP+DT’	0.3796	0.0417	0.064	1	2
2	‘3D+3D’	0.3569	0.0417	0	1	1
3	‘3D+DT’	0.5079	0.0417	0.4251	3	3
4	‘3D+CAM’	0.7121	0.0417	1	4	4
‘Bite off’ strategy (v = 0.5)						
1	‘HP+DT’	0.4694	0.1437	0.5142	3	3
2	‘3D+3D’	0.1396	0.0229	0.0000	1	1
3	‘3D+DT’	0.4684	0.1437	0.5127	3	2
4	‘3D+CAM’	0.7810	0.1437	1.0000	4	4
‘Mastication’ strategy (v = 0.5)						
1	‘HP+DT’	0.2220	0.0920	0.0708	1	1
2	‘3D+3D’	0.5434	0.1282	0.5253	3	3
3	‘3D+DT’	0.3860	0.0745	0.1574	1	2
4	‘3D+CAM’	0.7426	0.1983	1.0000	4	4

## Data Availability

The data presented in this study are available upon request from the corresponding author.

## Appendix A

**Table A1:** The values of the non-linearity factors.

Loading Points	No.	Non-Linear Criteria				
		R^2^	NC_L_	NC_S_	Std	RSS
V1	1	0.99115	0.99177	0.88554	69.6956	2881.57
	2	0.99987	0.99908	0.9863	14.9247	33.601
	3	0.99461	0.99597	0.94779	53.1685	1387.87
	4	0.98102	0.98775	0.83582	90.4759	6030.16
V2	1	0.99401	0.995947	0.913426	78.7707	1289.16
	2	0.99982	0.998693	0.981837	36.4377	37.803
	3	0.99797	0.99705	0.942395	36.774	566.777
	4	0.98336	0.990696	0.854617	99.6166	4257.44
V3	1	0.99566	0.994593	0.954078	93.8139	1021.06
	2	0.99419	0.994325	0.895573	98.0039	1206.66
	3	0.99579	0.996175	0.920074	63.1038	994.007
	4	0.98871	0.990925	0.863536	88.6193	2980.33
V4	1	0.99935	0.998716	0.995187	47.0266	106.461
	2	0.99206	0.994903	0.902241	64.7166	2109.09
	3	0.99775	0.997311	0.938626	64.7234	427.764
	4	0.97781	0.987629	0.824086	132.344	5203.2
V11	1	0.99597	0.995168	0.952817	84.3653	973.283
	2	0.99879	0.997108	0.949749	54.2114	251.912
	3	0.95768	0.975683	0.758979	151.4	11508.5
	4	0.97517	0.982872	0.799896	123.749	6922.29
V22	1	0.96455	0.986476	0.782089	276.328	5296.72
	2	0.9936	0.994923	0.891302	133.621	1065.39
	3	0.99327	0.993634	0.897892	111.108	1332.14
	4	0.98368	0.993541	0.852792	152.209	2910.07
V33	1	0.9639	0.959743	0.811918	263.367	5306.18
	2	0.99293	0.997154	0.892902	189.91	779.082
	3	0.99375	0.994721	0.914732	141.001	1058.97
	4	0.99049	0.99131	0.864647	170.376	1579.11
V44	1	0.99096	0.99681	0.889152	247.662	945.65
	2	0.94214	0.969829	0.728243	264.823	11342.1
	3	0.96567	0.991112	0.809016	273.422	4339.62
	4	0.9901	0.99605	0.875528	155.278	1386.35

**Table A2:** The durability estimates.

Loading Points	No.	$\bar{P_{2}}$	$\bar{M_{2}}$	$P_{2}$
V1	1	−6405.9523	−4530.0881	−348.640
	2	−256.3950	−315.9221	−39.496
	3	−101.6786	321.2428	303.366
	4	805.8142	703.4785	507.597
V2	1	776.3375	853.1463	624.282
	2	225.5808	221.1871	27.7192
	3	527.9545	467.2277	184.362
	4	1194.8662	838.7539	694.426
V3	1	1108.4774	1091.1320	219.489
	2	630.7553	1489.7861	698.392
	3	1183.0577	933.9553	419.943
	4	−716.2210	884.5791	456.108
V4	1	−83.3246	−3.0311	−251.060
	2	−271.8845	547.1103	460.318
	3	439.4961	552.4035	397.658
	4	1457.6006	1526.7168	1023.670
V11	1	856.8801	−928.0734	−475.330
	2	1020.3770	612.4722	298.827
	3	1226.2958	1487.9236	908.095
	4	1138.6096	1573.7441	731.402
V22	1	42,667.0120	40,731.1060	3622.410
	2	1457.3124	1235.4335	1162.850
	3	496.0120	1092.4758	838.074
	4	15,278.1010	17,598.1300	1569.220
V33	1	1888.5358	2964.5050	3434.930
	2	281.2275	4571.4109	2830.150
	3	1401.6739	959.0351	983.250
	4	2330.1384	2127.4319	1520.760
V44	1	9445.4123	3599.9183	3688.460
	2	3540.2154	2930.6892	2711.670
	3	5744.1242	2853.3027	4044.110
	4	3904.2487	3308.6874	1921.290
